# Control strategies to improve the low water quality of Souk-Ahras city

**DOI:** 10.1016/j.heliyon.2021.e07606

**Published:** 2021-07-17

**Authors:** Dhaouadi Mellahi, Ridha Zerdoumi, Assia Chaib

**Affiliations:** a Laboratory of Chemistry and Environmental Chemistry (L.C.C.E), Department of Chemistry, Faculty of Matter Sciences, University of Batna 1, 05000, Batna, Algeria; b Center for Scientific and Technical Research in Physico-Chemical Analysis, Bousmail, Tipaza, Algeria

**Keywords:** Waterborne diseases, Distribution systems, Water quality, Spearman correlations, EPANET

## Abstract

This work reports control strategies of the water quality in the city of Souk-Ahras (east Algeria). With the recent development, rapid population growth, and the consequences of climate change, the capacity of water supply reserves becomes more unpredictable in the long term. This has drastically affected the distributed water quantity. A correlation between bacteriological water analysis and the analysis of pollution indicative physicochemical parameters is developed to replace the slow bacteriological analysis, which takes more than two days, by directly accessible physicochemical analysis to anticipate the case-onset of waterborne diseases. A good correlation is found between different combinations of physicochemical pollution parameters: (Turbidity, Nitrates); (Turbidity, Active chlorine) (nitrates, active chlorine); (Ammonium, Chlorine) and (Turbidity, Ammonium) with Spearman rank coefficients of 0.8657, -0.8602 and -0.8531 -0.8227 et 0.7957 respectively. Besides, long term analysis (over several years) revealed a high correlation of more than 0.92 between the analysis of pollution indicative physicochemical parameters and bacteriological analysis. The EPANET software is used to simulate the hydraulic behaviour of the network system over an extended period within pressurized and pressure-deficient conditions. The simulation results of several supply scenarios of daily drinking water pressure in the city center area show that 62% of drinking water distribution system is supplied with a steep slope (80 m), 10% with unsatisfactory pressure and only 23% with acceptable pressure (1–80 m).

Therefore, the high working pressure at the mesh, and the interruptions of the water supply are factors that can lead to the occurrence of cross-connection cases. This diagnosis of the defects in the water supply system is combined with a statistical data analysis of physicochemical parameters to set up an effective sampling strategy that takes into account the frequency of analysis and the areas at risk to prevent the risk of waterborne diseases.

## 1 Introduction

According to the World Health Organization (World Health O., 2017a, World Health Organization, 2017), each year, diarrhoea kills around 525,000 children under five. Additionally, around 3.3 million symptomatic cases of hepatitis E are reported every year (Rein et al., 2012). Moreover, researchers have estimated around 1.3 to 4.0 million yearly new cases of cholera with 21 000 to 143 000 death cases worldwide (Ali et al., 2015). Among the main recommendations of the WHO to reduce the number of these cases is to provide clean drinking water and access to adequate sanitation and hygiene.

Waterborne diseases are mainly caused by connections between drinking water supply mains and the sewage pipe (cross-connections). The polluted water can be introduced through the cracks in the pipes or hydraulic equipment due to the pressure difference between the distribution system and surrounding contaminated sources. Cross-connections also cause chemical and biological contaminations of drinking water and the whole drinking water distribution infrastructure (tanks, pipes, etc.). Besides, microorganism detection in the network system can be the result of insufficient disinfection treatment. Indeed, the class of bacteria Bacilli and Clostridia spore-forming endows them an excellent resistance against desiccation and lack of nutrients (Laue et al., 2018; McKenney et al., 2013). In this regard, bacteria can reproduce in the distribution network under suitable conditions, such as: increased water temperature, presence of nutrients, decrease in the residual chlorine level, …etc (Setlow et al., 2017). This problem of water quality degradation is more or less specific to underdeveloped and developing countries and to countries with water resource deficiencies. Indeed, water shortages are increasing, especially with extensive development, which implies an increased demand for water in the 21st century (Balogun et al., 2017). Climate change can impact both the quantity and quality of water, and therefore the integrated management of dams, watersheds as well as domestic, industrial and irrigation water (Salami et al., 2015).

In this context, Algeria is not exempted. In fact, since 1975, the problem of waterborne diseases has affected several cities in Algeria with many cases of cholera and infectious hepatitis (ECDPC, 2018; Guechi, 1986). In the mid-80s, the country has recorded significant development in the realization of big hydraulic infrastructure, which has significantly reduced the cases of waterborne diseases.

According to a recent study on the consequences of climate change in North African countries (Hamed et al., 2018), reserves of surface water supply have decreased significantly over the last few decades. This has drastically affected the supplied water quantity leading to an intermittent water supply in the distribution mains.

The most notable preventive measures cited in the literature to fight against drinking water contamination are summarized below:

Mobilization of water resources and hygiene are the most effective ways to reduce microbial transmission according to Uprety (Uprety et al., 2020), Sojobi (2016) believes that providing clean water requires sustainable and efficient water management structure by public and private operators (Sojobi, 2016). Anticipation of climate change and multi-sectoral collaborations for integrated interventions to reduce waterborne disease cases in schools is needed (Cissé, 2019). For Galbraith et al., 1987, the collaboration between public health doctors and water engineers and scientists is necessary to reduce waterborne diseases (Galbraith et al., 1987).

Bacteriological analysis is commonly used to evaluate water contamination caused by network system failures. However, it is relatively slow and requires, at least two days, to confirm the results of possible water contamination. During the two days when the bacteriological analysis are not yet available, the contaminated water is still supplied to consumers, leading to severe consequences, even with an announces boil-water alert.

In this work, data of physicochemical and bacteriological analysis of water supply samples were collected over a long period of time. Then, the data were used in the development of a sampling strategy based on statistical calculations allowing us to determine the number of the samples that have to be analyzed on the one hand, and practical considerations to set the analysis frequencies and the most interesting areas on the other hand.

To overcome this problem, the study is focused on two key objectives:

1. The statistical exploitation of the available data of physico-chemical analyses and the failures noticed in the distribution network allowed us to set up an optimized sampling strategy based on three factors: the number of samples to be analyzed for each parameter, the choice of the analysis stations and the analysis frequency.
2. A procedure for alerting on the degradation of the bacteriological quality has been set up through the analysis of physicochemical parameters indicators of pollution which perfectly correlate with bacteriological parameters.

The correlation was also conducted between different pollution indicative physicochemical parameters, each parameter to other parameters in (X, Y) correlations, according to the Spearman rank correlation coefficients. Based on these correlations, cases of waterborne diseases can be predicted through simple control of pollution indicative physicochemical parameters instead of the time-consuming conventional bacteriological analysis.

## 2 Materials and methods

### 2.1 Study area description

The study is carried out in the city centre of Souk-Ahras. Souk Ahras is located in the North-East of Algeria. The drinking water distribution system of the city centre is supplied from three main reservoirs of 800 m^3^ each. The distribution system of the city centre is a mesh network with a steep slope.

### 2.2 Sampling strategy

The sampling strategy aims to improve the quality of the supplied water and to anticipate possible water quality degradation. This strategy was adopted using data of physicochemical analysis over a period of four years from 2012 to 2015.

Statistical calculation approach is used to determine the number of samples to be analysed for each parameter, considering the frequency of sampling in time and space.

The physicochemical parameters are divided into two main categories: global water quality parameters (hydrometric title, total alkalinity, salinity, pH, temperature...), and pollution indicative physicochemical parameters (nitrites, organic carbon, active chlorine, turbidity, bacteriological parameters, etc.) and parameters that are rarely analysed (heavy metals, specific pollution).

#### 2.2.1 Determination of the number of samples for analysis

The number of samples to be analysed for a given parameter can be determined taking into account the desired confidence interval. The precision and reliability of the statistical calculations depend on the number of performed analyses, provided that large data analysis is performed between 2012 and 2015. Variance estimate for simple random sampling (Kish, 1995; Wiegand, 1968) can be determined according to Eq. (1):

(1) $$\text{V}=\frac{{\text{S}}^{2}}{\text{n}}=\frac{{\text{d}}^{2}}{{\text{t}}^{2}},\;\text{So}\;\text{that}\;\text{n}=\frac{{\text{t}}^{2}.{\text{s}}^{2}}{{\text{d}}^{2}}$$

Where V: variance, S: standard deviation, d: margin of error (estimated according to the repeatability and reproducibility of the analytical method), n; the number of samples, t: Student for the desired confidence level.

The term student t (in the Student's t-test) can be determined using the desired confidence level p where p depends on the reliability of the analysis method (p is between 88 and 99.9%) according to Eq. (2):

(2) $$\text{t}=\sqrt{2}.{\text{erf}}^{-1}\left(\text{p}\right)$$

where erf^−1^ is the reciprocal error function.

### 2.3 Analysis methods

#### 2.3.1 Bacteriological control

Microbiological water analysis is mainly based on the concept of faecal indicator bacteria (Association, 2012). The main monitored parameters are faecal coliforms, total coliform bacteria include many members of the Enterobacteriaceae family, enterococci and heterotrophic aerobic and anaerobic bacteria (Lalancette et al., 2014; Shibata et al., 2004; Wang et al., 2015).

##### 2.3.1.1 Presumptive test

The presumptive test is a screening test of water samples for the presence and estimate of the concentration of coliform organisms. A series of lactose fermentation tubes are incubated at 35 ± 0.5 °C for 24 ± 2 h to prove the presence or absence of coliforms.

##### 2.3.1.2 Confirmatory test

In the confirmatory test, the presence of coliform organisms is confirmed when a gas phase is formed in any tube of the series. In this case, the water is considered unsafe.

##### 2.3.1.3 Final test

The final test is performed on a typical, well-isolated colony to reaffirm gas production in lactose, and to determine the morphology and the Gram reaction of the isolated form of a nutrient agar slant.

### 2.4 Analysis of water quality physicochemical parameters

The physicochemical analysis of the pollution indicators was carried out with a DR/2400 Portable spectrophotometer (HACH, Colorado, USA). The calibrations are pre-recorded in the memory of the spectrophotometer. After the calibration has been established, an appropriate reference sample is analysed. If the measurements exceed a 10% error, the instrument must be recalibrated. Periodic calibration of the spectrophotometer by standard solutions is checked before each analysis series. Physicochemical analysis was focused on pollution indicator parameters such as Organic carbon, dissolved oxygen, nitrites… (Mermillod-Blondin et al., 2005). The samples to be analysed for nitrates, nitrites and organic carbon were cooled down to 4 °C and analysed within 48 h. For spectrophotometric analysis of ammonium, indophenol blue reagents are added at the collection of the samples since the required time for complexation is a minimum of 6 h according to the US-EPEA 350.1 protocol (O'Dell, 1996). Residual chlorine is analysed using N,N-diethyl-p-phenylenediamine (DPD) (Uden and Miller, 1983). Organic carbon is analysed using the colorimetry method where the samples are oxidized in small safety sealed tubes containing the same reagents of the classical chemical oxygen demand (Micro Chemical Oxygen Demand). Either the amount of reduced chromium (trivalent) or the amount of unreacted dichromate (hexavalent) can be measured by spectrophotometric methods rather than titrimetry (Dharmadhikari et al., 2005).

### 2.5 Hydraulic system simulation

The simulation of the hydraulic behaviour of the distribution system was carried out using EPANET 2.0 software (Rossman, 2000). The model fidelity depends on the quality of the imput data. In addition to the data delivered by the Algerian Water Agency, precise data were obtained from the engineering office in charge of the diagnosis and study of Souk Ahras network system. The provided information are related to static and dynamic data such as the main and secondary network system (length, diameter, nature of the pipes...), altimetry of the nodes, pumps, tanks, control valve, non-return valve, the geographical distribution of the average annual consumption of the nodes, the average daily consumption of the various users (domestic, industrial, etc.).

The water supply for the city centre is surface water from the dam Ain Dalia. The water is treated by conventional treatment of surface water supply including pre-chlorination, alum coagulation/flocculation, filtration, and final disinfection with chlorine dioxide.

## 3 Results and discussion

### 3.1 Physicochemical and bacteriological quality assessment

#### 3.1.1 Physicochemical and bacteriological quality evolution in the network system

The city centre of Souk Ahras is supplied from surface water (Ain Dalia dam). Therefore, the physicochemical and bacteriological quality depends both on the quality of the raw water and the treatment process. The water from Ain Dalia dam is treated by the conventional process (coagulation, flocculation, decantation, filtration and chlorination). In the majority of positive cases of bacteriological analysis, we identified mainly total coliforms, thermotolerant coliforms, Escherichia coli and streptococci (faecal origin) responsible for the outbreak. Almost all the detected positive cases are caused by external contamination (cross-connections). Although most of the coliforms are not pathogenic, five cases of hospitalization are recorded with signs of typhoid fever, diarrhea and dysentery.

Diseases caused by certain microorganisms (bacteria, viruses, fungi, parasites) present in water are summarized in Table 1.

**Table 1:** Some microorganisms responsible for waterborne diseases.

Diseases	Causes	References
Typhoid fever	Salmonella Typhi bacteria	(Coulliette et al., 2013)
Diarrhoea	Viruses, bacteria and parasites from fecally contaminated water	(Ercumen et al., 2017)
Cholera	Bacterium Vibrio Cholerae	(Piarroux et al., 2011)
Dysentery	Bacillary dysentery: caused by bacteria. Amoebic dysentery: caused by amoebae.	(Wu et al., 2020)

**Figure 1:**
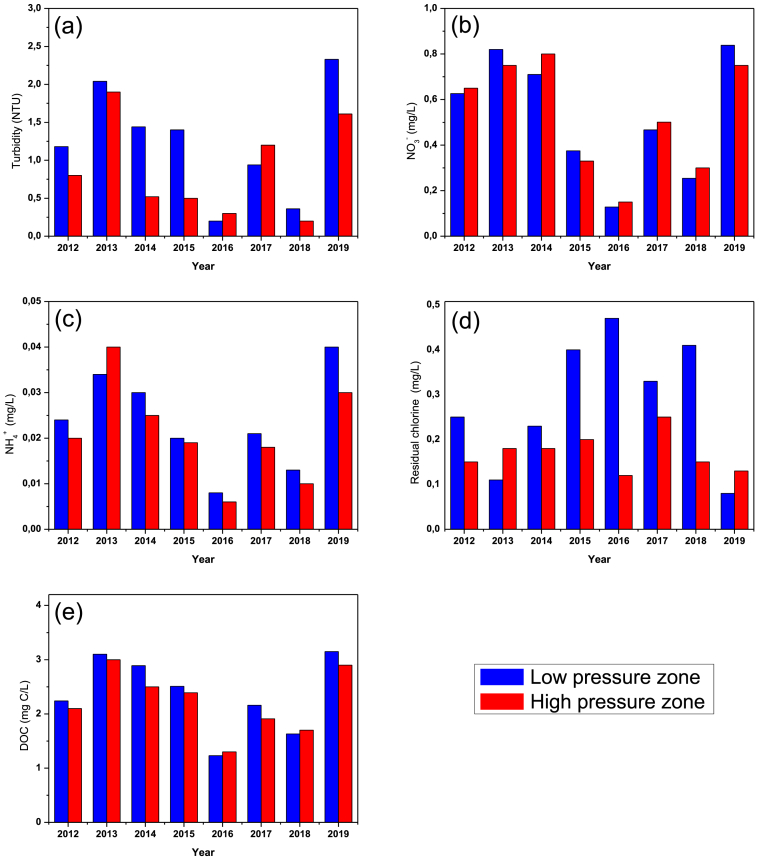
(a), (b), (c), (d) and (e) indicate respectively average concentrations of Turbidity, NO_3_, NH_4_^+^, residual chlorine and DOC in the low and high-pressure zone of Souk Ahras city center areas.

For the physicochemical analyses, Figure 1 shows the average annual concentrations of the main physicochemical pollution indicator parameters. Nitrites are not included in the figure because their annual average concentration tends towards zero. All parameters are below the Algerian and WHO standards (Table 2), except for dissolved organic carbon because the surface water treatment plant does not use activated carbon to remove dissolved organic matter. A variation of physicochemical parameters from one year to another was observed. Indeed, contrary to groundwater, the quality of surface water frequently fluctuates, depends on several factors such as flow rate of floods and velocity in the watershed, precipitation frequency, dam level, temperature etc.

**Table 2:** Main Algerian and WHO drinking water standards used in this study.

Parameters	Algerian drinking water standards (OJAR, 2011)	WHO drinking water standards (World Health O., 2017a, World Health Organization, 2017)
Bacteriological parameters		
Total coliforms (n/100 mL)	10	**00**
Fecal coliforms (n/mL)	00	**00**
Fecal Streptococci (n/mL)	00	**00**
Enterococci (n/100 mL)	00	**00**
Escherichia Coli (n/100 mL)	00	**00**
Sulphite-reducing bacteria including spores (n/20 mL)	00	**00**
Salmonella	Absence	**Absence**
Physicochemical parameters		
Ammonium (mg/L)	0,5	**0,5**
DOC (Oxidizable matters) mg O_2_/L	05	-
Nitrates (mg/L)	50	**50**
Nitrites (mg/L)	0,2	**0,2**
Turbidity (NTU)	**05**	**05**

To study the variation of physicochemical parameters in the network, we calculated the annual average concentration of each parameter for two areas of the Souk Ahras city center network (high-pressure zone and low-pressure zone). The variation of the parameters in the zones is not very remarkable, except for the residual active chlorine and turbidity. The concentration of residual active chlorine of low-pressure zone is higher than the high-pressure zone. This can only be explained by the long distance between the supply tanks and the high-pressure zone, unlike the low-pressure zone, which is located right next to the distribution tanks.

#### 3.1.2 Recurring leaks and waterborne diseases

The evolution of leaks has been monitored at the level of distribution mains, transmission mains and household connections from 2012 to 2019. Usually, leaks are registered at the Operational Telephone Reception Center (OTRC). The OTRC operates 24/7/, and its main objective is to receive and respond to complaints in the event of water cuts, leaks, suspicions about the quality of the water, commercial complaints, etc. When leaks are not apparent, identification is achieved through ultrasonic leak detectors by specialized maintenance teams. The estimation of leaks is done indirectly by comparing the distributed quantity measured by sub-meters, and the consumed quantity by the individual meters of the consumers.

**Figure 2:**
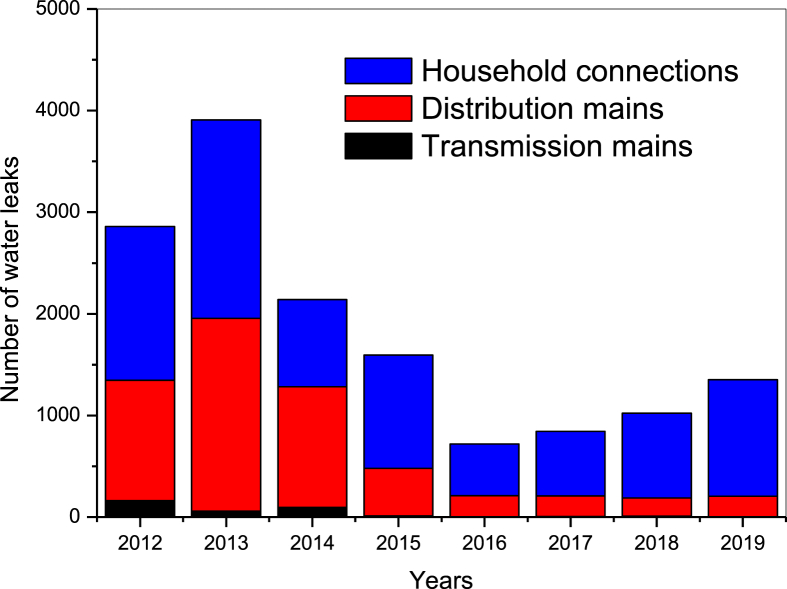
Evolution of leaks in different types of water supply network.

The rehabilitation of the city's distribution system has affected practically all equipment and structures namely: Transmission mains, water supply network, household water connection, network protection devices (pressure reducing valves, isolation valves, control valves...). This rehabilitation has gradually reduced the number of total leaks from 3909 in 2013 to 720 in 2016 (Figure 2). On the other hand, between 2012 and 2013, an increase in the number of leaks has been noticed. This can be explained by the beginning of the rehabilitation construction work, leading to more leaks. In this evolution, the number of leaks in the transmission main has been zero since 2015.

#### 3.1.3 Waterborne diseases and low water quality evolution

**Figure 3:**
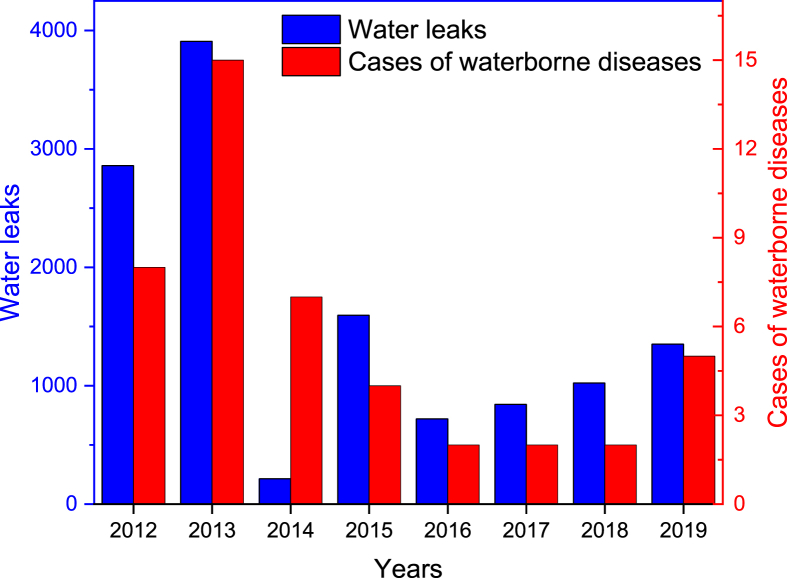
Relationship between waterborne diseases and leaks.

Figure 3 clearly shows a proportional relationship between the number of leaks and the cases of waterborne diseases. This explains why the cases of waterborne diseases from 2012 to 2019 are related mainly to the distribution network system. During 2014, an unusual increase in the number of waterborne diseases is observed. Indeed, during network system rehabilitation, an old network that should be abandoned was still connected. The latter was defective and became a source of contamination, especially during water supply suspension periods. The identification of this contamination took a long time, which explains the unusual increase in the cases of waterborne diseases compared to the recorded number of leaks in this period.

The reduction in the number of leaks to 720 in 2016 has practically eradicated the cases of waterborne diseases. The low water quality led to the hospitalization of two cases suspected to be suffering from water contamination on 45 cases recorded between 2012 and 2019. Despite the significant increase in analysis frequency from 2012 to 2019, a significant decrease in waterborne disease cases was noticed. The disease cases recorded from water transmission were limited to a few districts and are due to external constraints of the distribution network. However, other observed internal conditions during this investigation are originated from the appearance of poor water quality due to the interaction of the distributed water and biofilms (Carter et al., 2000). This situation has been observed in old distribution system network subject to special conditions such as discontinuous drinking water supply and fluctuation in the feed rate (Fish et al., 2017). These drinking water supply conditions led probably to the release of bacteria injured and/or developed at the internal walls of the pipes. The rehabilitation of the distribution network has given total satisfaction to the majority of districts. For other districts, the conditions that led to the deterioration of the supplied water quality are mainly the same. The presence of diverse road networks, transportation, sanitation, electricity and many industrial and service activities, in an anarchic and precarious situation made it very difficult to rehabilitate these districts in adequate conditions.

**Figure 4:**
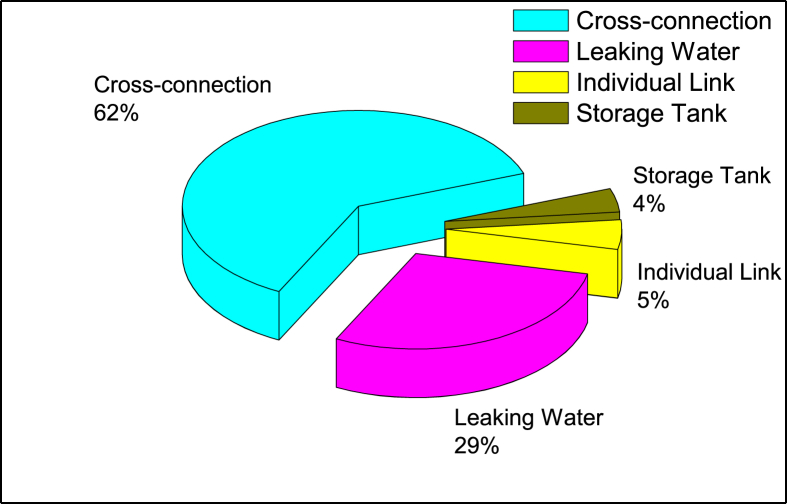
Cases of waterborne disease outbreaks caused by water supply system deficiencies.

Identifying the contamination origin(s) in the distributed water will be the basis for making any required adjustments to the rehabilitation plan of the distribution system. Figure 4 showed different categories of constituent equipment in the distribution system which have led to outbreaks. It indicates that cross-connections account for 62% (28 contamination cases) while the leaking mains account for 29% (13 contamination cases). These two main contamination sources are associated with discontinuous supply in the distribution network. The household connection and out-of-control customer interventions accounted for 5% (3 contamination cases). Contamination of storage tanks represents 4% (2 contamination cases). For cross-connections, the vast majority of cases were located in old buildings where the secondary drinking water distribution network passes near the sewerage network in the basements.

### 3.2 Strategy for controlling water quality degradation

In order to develop an adequate approach to control bacteriological water quality degradation of Souk Ahras city center, we have, firstly, exploited the availability of sufficient physicochemical analysis information to determine the number of analysis to be performed for each physicochemical parameter by statistical calculations. In addition, the distribution network system diagnosis allowed us to identify the areas with high risk of bacteriological contamination. By synergy of these two valuable information, we have managed to establish an optimized and effective sampling program that takes into account the frequency and the spatiotemporal sampling.

#### 3.2.1 Sampling strategy: determination of the number of samples

Using Eq. (1) presented above, the required number of samples to be analysed can be determined using analytical data from the 2012–2017 period. The number of samples n to be analysed as shown in Table 3 does not reflect the variability in absolute terms, since it does not take into consideration space and time. However, when the proportionality ratio R is calculated, it can provide an important indication of the frequency and the importance of the parameter(s) that have to be analysed frequently, and therefore allows for more elaboration of the sampling program.

**Table 3:** Determination of the number of samples.

Parameter	DOC	Turbidity	${\text{NH}}_{4}^{+}$	${\text{Cl}}_{2}$	${\text{NO}}_{2}^{-}$	${\text{NO}}_{3}^{-}$
Average concentration (mg/L)	2.17 mg/L	1.02 NTU	0.02	0.25	0.0006	0.42
Standard deviation (S)	1.07	0.87	0.010	0.18	0.020	0.29
margin of error (d)	0.2	0.12	0.001	0.02	0.001	0.01
desired confidence level (p)	95%	95%	95%	99%	98%	90%
Student (t)	1.96	1.96	1.96	2.58	2.33	1.64
samples number (n)	110	202	384	539	2172	2262
Number of proportional analysis (R)	1	2	3	5	20	21

Interestingly, nitrates and nitrites are around 1/20 of the total DOC. On the other hand, the number of chlorine analysis is typically low (1/5 of the DOC), since active chlorine analysis has the same or even more importance than the analysis of nitrates and nitrites. Active chlorine is a strong indicator of pollution emergency when sudden residual active chlorine decrease in the distribution main is observed.

#### 3.2.2 Diagnosis of the water-supply system in the city centre

The simulation of the hydraulic behaviour of the network system was carried out for the city centre of Souk-Ahras before and after the rehabilitation of the network system (2015–2019).

The simulation results of several supply scenarios of daily drinking water pressure in the city center area show that 62% of drinking water distribution system is supplied with a steep slope (80 m), 10% with unsatisfactory pressure and only 23% with acceptable pressure (1–80 m). The results also show that the flow velocity is between 0.7 and 1.5 m/s.

**Figure 5:**
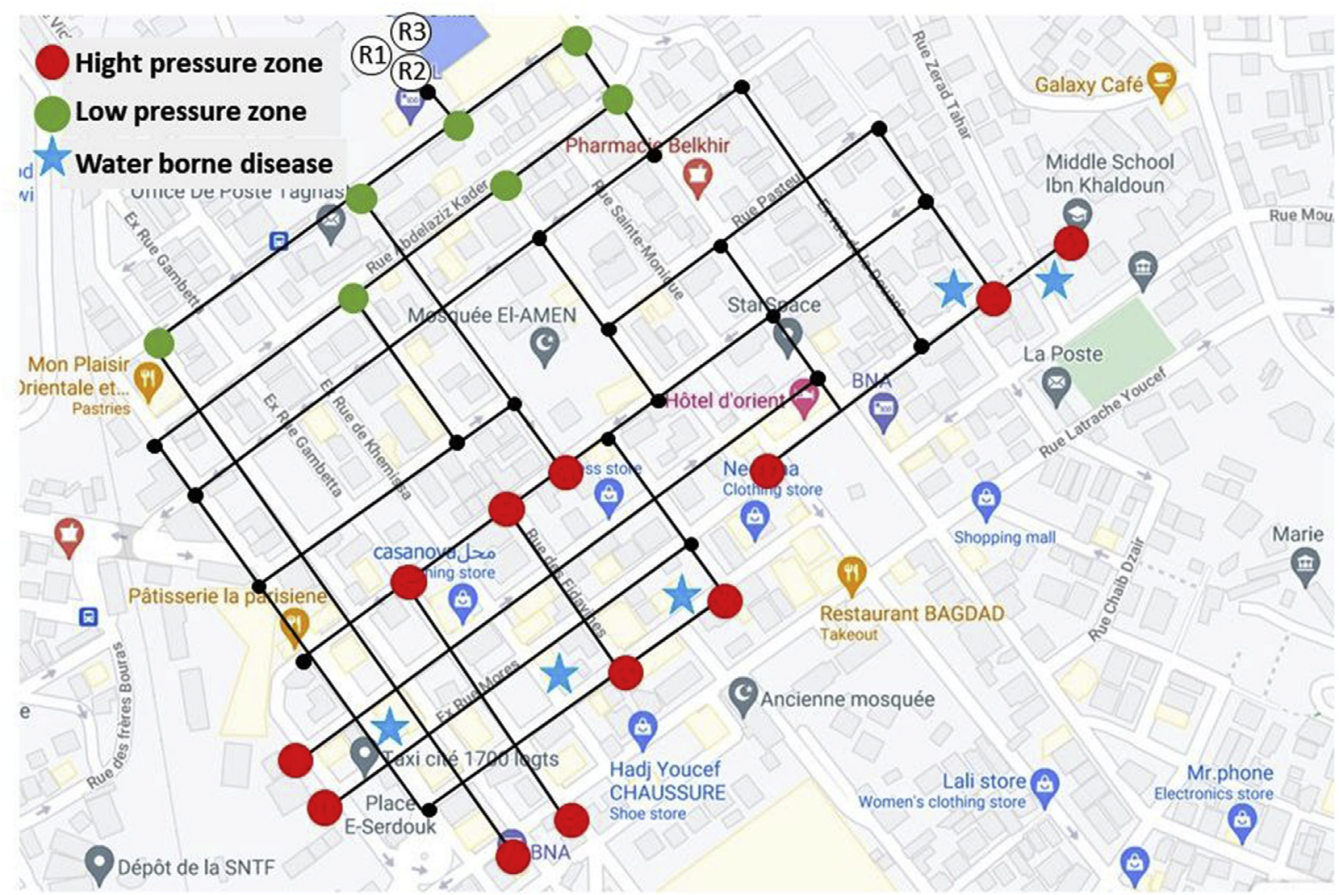
Simulation of the hydraulic behaviour of the distribution network with high and low-pressure zones in the city centre. The blue stars correspond to the real distribution waterborne disease area.

This simulation allowed us to identify high-pressure nodes and low-pressure zones when the network (Figure 5) is under pressurised and pressure-deficient conditions (Kurek and Ostfeld, 2013; Rossman, 2004).

Usually, the zone of low-pressure meshes can be associated with high probability risk points of waterborne diseases due to the back-siphonage during interruption periods of drinking water supply. Interestingly, the results show that all the reported cases of waterborne diseases are associated with the zone of high-pressure meshes. This finding can be explained by the long interruption periods of drinking water supply and the multiple leak cases at individual household connections located in the zone of high-pressure meshes.

The results of this part, allowed us to focus the physicochemical analysis frequently concerning active chlorine and nitrites in the zones with high pressure and in particular during the resumption of water distribution after a more or less prolonged period of interruption.

#### 3.2.3 Correlation between pollution, physicochemical parameters and bacteriological analysis

Bacteriological analyses are time-consuming where the results can be available only after 24 and 48 h to identify and confirm contaminated sites. Physicochemical analysis of pollution parameters were of great utility to elucidate contamination risks in much shorter periods of time compared to bacteriological analysis. Physicochemical analysis of pollution parameters has improved our ability to anticipate the contamination risk.

To make the correlation between the different physicochemical parameters reliable and representative, we have taken into consideration only the analyses of the parameters performed together for the same sample. For active chlorine, which is analyzed with a very high frequency, the analytical results considered for the correlation are those that were analyzed simultaneously with the other parameters for a given sample.

In the majority of cases, changes of the physicochemical pollution parameters such as nitrites, nitrates, ammonium, dissolved organic carbon, turbidity and residual chlorine were very reliable indicators. Furthermore, the emergence of nitrites and high active chlorine consumption in analysed waters samples shows a good correlation with positive bacteriological analysis. Nitrites are an important intermediate in the metabolism of nitrogen compounds. They are part of the nitrogen cycle between ammonia and nitrates.

Nitrites are unstable intermediates and can be easily oxidized to nitrates, especially in the presence of active chlorine. The chemical reduction of nitrates to nitrites (Equation 3) cannot take place spontaneously and necessarily requires the presence of bacteria.

(3) NO2- + 12O2→ NO3-

The presence of nitrites in main water may also indicate the aerobic autotrophic catalytic oxidation of ammonia to nitrite (Equation 4) by a new group of bacteria (Francis et al., 2005; Joicy et al., 2019).

(4) NH4++32O2→NO2-+2H++H2O

Also, the turbidity that can seep into the distribution network could provide the bacteria with a physical refuge and immunity against chlorination (Lynch et al., 2014; Tang et al., 2021).

**Figure 6:**
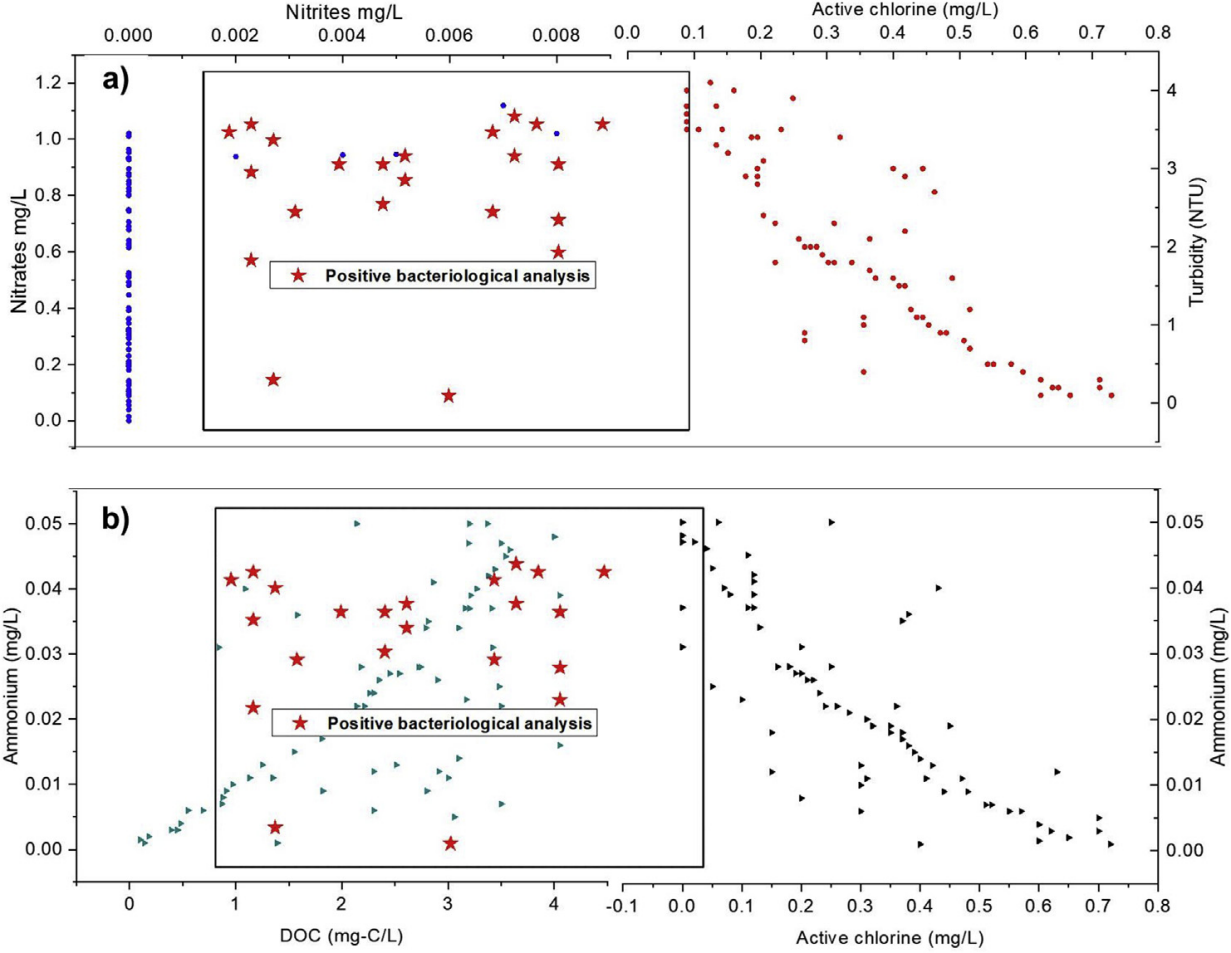
Correlation between pollution parameters and bacteriological analysis.

Figure 6 illustrates both the bacteriological analysis of positive cases versus six pollution indicative physicochemical parameters and four pairs of the Spearman correlations. The figure is partitioned into two parts (a) and (b) to directly read the concentrations of the different physicochemical parameters according to positive cases of the bacteriological analysis.

From Figure 6, we could see that the turbidity is more than 3 NTU while the corresponding residual chlorine is zero. With the presence of nitrite, about 92% of the bacterial analysis are positive. However, when the active chlorine concentration is above 0.3 mg/l, and even with turbidity greater than 3 NTU, bacteriological analysis has always been negative.

According to Table 4, a good correlation was found between the following pairs of physicochemical pollution parameters: (Turbidity, Nitrates); (Turbidity, Chlorine Active) and (nitrates, Chlorine active); (Ammonium, Chlorine) and (Turbidity, Ammonium) with Spearman rank coefficients of 0.8657, -0.8602, -0.8531, -0.8227 and 0.7957 respectively. For other pairs, the Spearman correlation is medium to low. Three significant correlations are retained, firstly, the correlation between turbidity and active chlorine with a negative Spearman coefficient of -0.8602, indicates that the increase in turbidity by cross-connection or release of biofilm results in the consumption of active chlorine. Secondly, the correlation between ammonium and active chlorine is also negative. During the long retention time from the water production to the consumer, the excess of chlorine causes progressive degradation of ammonium, especially when it is present in small quantities, such as in the surface water supply for the city centre. For the third correlation, although Spearman's coefficient is lower than the previous correlations (-0.4672), the appearance of nitrites is only possible in the absence of active chlorine. The treatment using sufficient amounts of chlorine to maintain the residual chlorine at acceptable levels (below 0.6 mg/l) is used to protect the water from eventual contamination. In addition, turbidity, which is directly perceptible by the consumer, can be a warning of possible water quality degradation. Therefore, it is important to encourage people to sensitize using sodium hypochlorite for disinfection, in case of suspecting the water quality (Sojobi et al., 2015).

**Table 4:** Spearman correlations of the pollution physicochemical parameters. Correlation is significant at the <0.05 level.

Parameters	Turbidity	Nitrites	Nitrates	Active chlorine
Turbidity	1			
Nitrites	0.4164	1		
Nitrates	0.8657	0.3684	1	
Active chlorine	-0.8602	-0.4672	-0.8531	1

## 4 Conclusion

The diagnosis of the drinking water distribution network of the city of Souk-Ahras has been studied to reduce the cases of waterborne diseases. To achieve this goal, a correlation strategy based on statistical calculations between physicochemical and bacteriological analysis has been developed based on a set of data from 2012 to 2019. In addition, a correlation between the physicochemical pollution indicator parameters and bacteriological parameters for early prediction of possible pollution of the distributed water has also been developed. This helps the intervention before confirming the contamination with bacteriological analysis, to protect the consumers against potential contamination risks.

The main cause of distributed water contamination is related to extended period of interruption in the water supply system, where the low-pressure meshes draw in contaminants (back-siphonage).

To remedy this situation, a spatio-temporal and frequency sampling plan is set up based on physicochemical parameters and statistical analyses on the one hand and diagnosis of the distribution system carried out by EPANET on the other.

Nitrite, nitrate and active chlorine are the must-to-be analysed in large numbers compared to other parameters. This is further strengthened by a good correlation between different combinations of physicochemical pollution parameters: (Turbidity, Nitrates); (Turbidity, Active chlorine) (nitrates, active chlorine); (Ammonium, Chlorine) and (Turbidity, Ammonium) with Spearman rank coefficients of 0.8657, -0.8602 and -0.8531 -0.8227 and 0.7957 respectively. In addition, the degradation of the physicochemical pollution indicator parameters are in perfect correlation with the positive cases of bacteriological analysis. This correlation means that the removal of the residual active chlorine and the formation of nitrites indicate that more than 92% of the bacteriological analysis are positive. This allows the monitoring of the evolution of water quality instantly. This result allows reducing the reaction time to deal with the pollution caused by network system failures since bacteriological analysis requires at least two days to determine whether the water is contaminated or not.

## Declarations

### Author contribution statement

Dhaouadi Mellah, Ridha Zerdoumii & Assia Chaib: Performed the experiments; Analyzed and interpreted the data; Wrote the paper.

### Funding statement

This research did not receive any specific grant from funding agencies in the public, commercial, or not-for-profit sectors.

### Data availability statement

Data included in article/supplementary material/referenced in article.

### Declaration of interests statement

The authors declare no conflict of interest.

### Additional information

No additional information is available for this paper.
